# Novel Clinical mNGS-Based Machine Learning Model for Rapid Antimicrobial Susceptibility Testing of Acinetobacter baumannii

**DOI:** 10.1128/jcm.01805-22

**Published:** 2023-04-06

**Authors:** Xuejiao Hu, Yunhu Zhao, Peng Han, Suling Liu, Weijiang Liu, Cong Mai, Qianyun Deng, Jing Ren, Jiajie Luo, Fangyuan Chen, Xuefeng Jia, Jing Zhang, Guanhua Rao, Bing Gu

**Affiliations:** a Department of Laboratory Medicine, Guangdong Provincial People's Hospital (Guangdong Academy of Medical Sciences), Southern Medical University, Guangzhou, China; b Genskey Medical Technology Co., Ltd., Beijing, China; c Department of Critical Care Medicine, Guangdong Provincial People's Hospital (Guangdong Academy of Medical Sciences), Southern Medical University, Guangzhou, China; d Tianjin Medical University General Hospital, Tianjin, China; Medical College of Wisconsin

**Keywords:** rapid antimicrobial susceptibility testing, antimicrobial resistance, clinical metagenomics, machine learning, *Acinetobacter baumannii*

## Abstract

Multidrug-resistant (MDR) bacteria are important public health problems. Antibiotic susceptibility testing (AST) currently uses time-consuming culture-based procedures, which cause treatment delays and increased mortality. We developed a machine learning model using Acinetobacter baumannii as an example to explore a fast AST approach using metagenomic next-generation sequencing (mNGS) data. The key genetic characteristics associated with antimicrobial resistance (AMR) were selected through a least absolute shrinkage and selection operator (LASSO) regression model based on 1,942 A. baumannii genomes. The mNGS-AST prediction model was accordingly established, validated, and optimized using read simulation sequences of clinical isolates. Clinical specimens were collected to evaluate the performance of the model retrospectively and prospectively. We identified 20, 31, 24, and 3 AMR signatures of A. baumannii for imipenem, ceftazidime, cefepime, and ciprofloxacin, respectively. Four mNGS-AST models had a positive predictive value (PPV) greater than 0.97 for 230 retrospective samples, with negative predictive values (NPVs) of 100% (imipenem), 86.67% (ceftazidime), 86.67% (cefepime), and 90.91% (ciprofloxacin). Our method classified antibacterial phenotypes with an accuracy of 97.65% for imipenem, 96.57% for ceftazidime, 97.64% for cefepime, and 98.36% for ciprofloxacin. The average reporting time of mNGS-based AST was 19.1 h, in contrast to the 63.3 h for culture-based AST, thus yielding a significant reduction of 44.3 h. mNGS-AST prediction results coincided 100% with the phenotypic AST results when testing 50 prospective samples. The mNGS-based model could be used as a rapid genotypic AST approach to identify A. baumannii and predict resistance and susceptibility to antibacterials and could be applicable to other pathogens and facilitate rational antimicrobial usage.

## INTRODUCTION

Multidrug-resistant (MDR) bacteria have been acknowledged as one of the most important public health problems. Inappropriate drug usage promotes the emergence and spread of MDR bacteria as infectious diseases become more prevalent, and the limited number of drugs can no longer meet the demand for treatment (1). The exploration of rapid antimicrobial susceptibility testing (AST) based on molecular methods represents an attractive approach for the early detection of MDR bacteria. However, due to differences in genotype and phenotype, determining exactly how to use molecular data to generate accurate AST results has been difficult for scientists (2, 3). Here, we utilized a rapid AST prediction model to obtain accurate antimicrobial susceptibility results, and this model was successfully applied to Acinetobacter baumannii.

MDR A. baumannii strains have been designated as a global threat to human health by the WHO. Resistance arises in MDR A. baumannii through a variety of mechanisms (4, 5), and its virulence factors are also complicated, resulting in high mortality (6). In China, the rate of immediate resistant A. baumannii increased from 31.0% to 71.2%, and the rate of meropenem resistance increased from 39.0% to 71.9% from 2005 to 2021 (7). Thus, resolving MDR A. baumannii infection and preventing its spread are critical (8). In addition, carbapenem-resistant *Enterobacteriaceae* (CRE), methicillin-resistant Staphylococcus aureus (MRSA), and vancomycin-resistant *Enterococcus* (VRE) present these challenges (9–11).

Broth microdilution, the “gold standard” for susceptibility testing, takes 16 to 24 h after a positive culture, leading clinicians to miss the optimal administration period. Many promising approaches have been established for phenotypic detection. For instance, the rapid ResaImipenem/*Acinetobacter* NP test detected carbapenem resistance within 2.5 h (12). For matrix-assisted laser desorption ionization–time of flight mass spectrometry (MALDI-TOF MS)-based rapid AST, the concordance rates of its MIC were 77.1% and 70.1% (13). Whole-genome sequencing (WGS) has emerged as a powerful tool for rapidly predicting many antimicrobial phenotypes for a bacterial strain (14). WGS-based AST models have been used to predict antimicrobial phenotypes in Staphylococcus aureus (15), Mycobacterium tuberculosis (16), Neisseria gonorrhoeae (17), and *Enterobacteriaceae* (18). To date, only one study (19) has reported a WGS prediction model to predict antimicrobial phenotypes based on 14 MDR A. baumannii strains, and the prediction consistency was unsatisfactory for determining resistance to antibiotics, such as ampicillin (57.1%), ceftazidime, and nalidixic acid (71.4%). The aforementioned AST techniques, including WGS, still rely on bacterial enrichment and are time-consuming; thus, they often do not meet clinical demands. These situations highlight the urgent clinical necessity of developing rapid and accurate AST strategies without culture processes.

Recently, the development and application of metagenomic next-generation sequencing (mNGS) have greatly aided in the clinical diagnosis of infectious diseases, enabling the accurate identification of virtually all potential pathogens in a clinical sample as well as providing ancillary genomic information about antibiotic resistance genes (ARGs). Currently, there is a lack of methods and tools with superior performance that directly predict antibiotic resistance and susceptibility from mNGS data.

**FIG 1:**
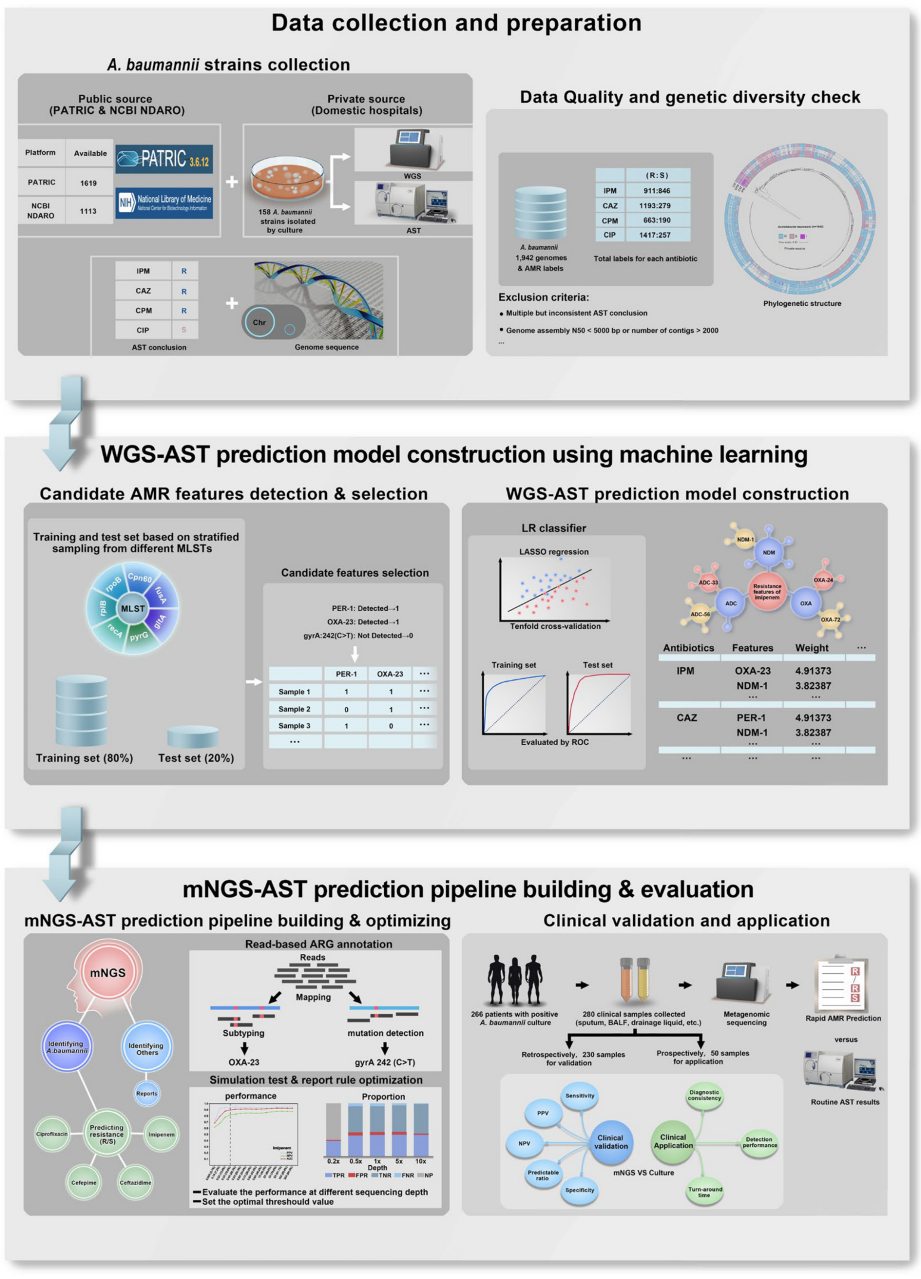
Study overview. A gene sequencing-based AST prediction model that directly used metagenomic sequencing of clinical specimens to identify antibiotic resistance determinants by aligning short-read sequence to a curated ARG reference database and to infer the susceptibility and resistance of A. baumannii. (Top) A large number of strain genomes of A. baumannii with associated AST conclusions were collected from the public PATRIC and NCBI NDARO databases, as well as domestic hospitals, and some strains with low quality were strictly filtered according to a customized criterion. A phylogenetic tree was generated to explore the genetic diversity and representativeness of these collected strains simultaneously. (Middle) After quality control, all strains were randomly divided into training and test sets at a ratio of 8:2 based on stratified sampling from different MLSTs to generate a WGS-AST prediction model using LASSO regression and to verify the performance, during which the key genetic features significantly associated with antibiotic resistance were identified. (Bottom) A read-based ARG test pipeline that could be used to simultaneously detect the presence or absence and SNPs/short indels of genes was generated and optimized through read simulation experiments of clinical isolates. Given the influence of the amount of sequencing data, reporting rules were specifically formulated, and optional thresholds were set up. A small batch of clinical specimens was also collected from domestic hospitals to validate the performance of the mNGS-AST prediction model retrospectively and prospectively. PATRIC, Pathosystems Resource Integration Center; NDARO, National Database of Antibiotic Resistant Organisms; IPM, imipenem; CIP, ciprofloxacin; CAZ, ceftazidime; CPM, cefepime; AMR, antimicrobial resistance; MLST, multilocus sequence typing; LR, LASSO regression; AST, antimicrobial susceptibility testing; ARG, antibiotic resistance gene; PPV, positive predictive value; NPV, negative predictive value.

In this study, we proposed a machine learning-based method combined with mNGS for the rapid prediction of antimicrobial phenotypes using A. baumannii as an example to explore rapid AST in clinical microorganisms. The work described in this article consists of three sequential steps (Fig. 1). First, the initial model was trained on susceptibility tests and WGS data, and the key genetic features significantly associated with antibiotic resistance were selected using least absolute shrinkage and selection operator (LASSO) regression. Second, based on these key genetic features, we accordingly established and optimized an mNGS-based AST prediction model directly using clinical specimens to identify antibiotic resistance determinants and infer the antibacterial phenotype. Finally, 230 retrospective clinical specimens and 50 prospective samples were collected to validate the mNGS-AST prediction model.

The strategy used for this proposed model is not only applicable to the analysis of A. baumannii; other MDR isolates could also be analyzed with a similar approach to develop rapid AST prediction models. Given the widespread use of mNGS in clinical practice, this method could serve as a reliable basis for early clinical decision-making regarding therapeutic schedules (within a day) to achieve the goal of early treatment and precise medication.

## MATERIALS AND METHODS

### Ethics statement.

The participants involved in this study were in accordance with the guidelines of the Research Ethics Committee of the Guangdong Provincial People’s Hospital, Guangdong Academy of Medical Sciences (KY-N-2022-003-03). All participants provided oral informed consent.

### Collection of A. baumannii genomes.

Whole genomes from A. baumannii isolates with AST data were collected from public databases and domestic hospitals. A customized criterion formulated by referencing the NCBI genome exclusion rules was applied to remove low-quality genomes (see Section 1 in the supplemental material). A total of 1,942 A. baumannii whole genomes remained after this filtering step and were used to develop a gene sequencing-based ARG test pipeline, including 1,784 genomes from the PATRIC (20) and NCBI NDARO databases (21) and 158 genomes from domestic hospitals (see Table S1 in the supplemental material). The following four clinical first-line antibiotics were used: imipenem (IPM), ceftazidime (CAZ), cefepime (CPM), and ciprofloxacin (CIP) (see Table S2 in the supplemental material).

### Phylogenetic analysis and multilocus sequence typing.

Phylogenetic trees were generated to explore genetic diversity within the population of A. baumannii isolates as previously described (22–25). The multilocus sequence typing (MLST) was performed for each strain by directly aligning genome contig sequences to all alleles of seven housekeeping genes of A. baumannii using BLASTN (version ncbi-blast-2.9.0+) and comparison with the allele profiles (see Section 2 in the supplemental material).

### Curation of the ARG reference database.

The ARG reference database included all nucleic acid sequences from the CARD database (26) and two fluoroquinolone resistance-related wild-type genes, *gyrB* and *parC*, in A. baumannii from the NCBI gene database. By referring to the MEGARes database (27), each gene was acyclically annotated with six hierarchical levels, which enabled us to implement the lowest common ancestor (LCA) annotation strategy (28). All pairwise genes in the reference database were aligned using MUSCLE software (version 3.8.31) to calculate the similarity. More details can be found in Section 3 of the supplemental material. Cluster analysis was conducted based on the sequence similarity between any two members within the *ADC* family, and a cluster tree was plotted with the positive predictive value (PPV) annotation of CAZ, CPM, and IPM for A. baumannii. Hence, *ADC* subfamilies, such as *ADC-30-like* and *ADC-240-like*, were defined (see Fig. S1 in the supplemental material).

### Using machine learning to screen resistance markers.

Genome contig sequences were aligned to the curated ARG reference database using BLASTN to test ARGs and single nucleotide polymorphisms (SNPs)/indels of ARGs by parsing m0-format alignments with a self-built Perl script program (more details are available in Section 4 of the supplemental material).

For a pair of pathogen and antibiotic combinations, the association between genomic features and AST results was explored using a generalized linear model, a random forest model, and a LASSO regression model (29). Compared with the generalized linear model and the random forest model, the LASSO regression model, which effectively addressed the issues of collinearity and overfitting, demonstrated the same good performance with an area under the concentration-time curve (AUC) value over 0.9 (see Table S3 in the supplemental material); therefore, this model was ultimately utilized to evaluate the contribution of each screened critical ARG to the resistance phenotype and to calculate the ARG coefficients (weight).

All strains were stratified according to MLST classification and randomly divided into training and test sets at a ratio of 8:2 for the training and validation of the model. During the process of model training, the genetic data were transformed into a 0 to 1 type matrix, in which 1 indicated that ARG or ARG variation was detected. The LASSO regression model was constructed using the R glmnet package, and 10-fold cross validation was applied. The AUC was calculated to evaluate the performance of each model. In addition, the weight coefficients of the ARG family to which the screened key ARG subtype belonged were calculated by the following formula:

$$\mathrm{Genefamily}\_W=\frac{\sum_{i=1}^{j}A\mathrm{MR}\_\mathrm{feature}\_N_{i}\text{*}\mathrm{AMR}\_\mathrm{feature}\_W_{i}}{\sum_{i=1}^{j+k}A\mathrm{MR}\_\mathrm{feature}\_N_{i}}$$

where *AMR_feature_N* indicates the number of samples in which key ARG subtype or ARG variation features were detected, and *AMR_feature_W* refers to the weight coefficient of the detected key ARG subtype or ARG variation features. *j* indicates the number of detected key ARG subtypes or ARG variation features. *k* indicates the number of all detected nonkey ARG subtypes within the family in the training data set.

The LASSO regression classifiers were trained using random subsamples of different sizes with 10 repetitions in the training data set to investigate how prediction performance was affected by the number of samples. For each model of the pathogen/antibiotic combination, the AUC value was recorded in 10 of the 10-fold nested cross-validation. The AUC value distribution was utilized to evaluate whether the sample size was sufficient.

### Read-based ARG test pipeline and antibiotic resistance prediction.

The ARG annotation and subtyping pipeline was developed to accurately characterize ARG subtypes by directly aligning unassembled mNGS reads to the ARG reference database using BLASTN, and a greedy algorithm and LCA approach were applied to filter out the false positive ARGs (see Fig. S2 as well as Section 5 in the supplemental material). Next, the score index, an interpretation indicator, was further defined and calculated based on identified ARG subtype/variation features or ARG family with weight coefficients for each strain using the formula below. The maximal Youden index was used to estimate the cutoff value for reporting resistance using receiver operating characteristic (ROC) analysis through short-read simulation experiments based on the training data set.

$$\mathrm{Score}=\overset{n}{\underset{i=1}{\sum }}G\mathrm{enefamily}\_W_{i}\;||\;\mathrm{AMR}\_\mathrm{feature}\_W_{i}$$

*AMR_feature_W* refers to the weight coefficient of the detected key ARG subtype or ARG variation features. *Genefamily_W* indicates the weight coefficient of the ARG family to which the detected key ARG subtype belongs. *n* indicates the number of detected ARG subtypes/ARG variation features or ARG families. The coefficient of the ARG family is only added if the coefficient of the ARG subtype is not >0.

For mNGS data, the species source of detected ARGs needed to be inferred before calculating the score index. Here, the species attribution of ARGs was mainly determined based on whether the calculated copy number of ARGs was within the normal range for the assumed affiliation of the gene-species (Fig. S2). If the calculated copy number was normal, we accepted the null hypothesis; otherwise, it was rejected.

### Simulation experiment.

Short-read sequences obtained from clinical isolates were simulated using ART software (version 2.5.8) (30) to conduct read-based ARG testing for the following purposes.

(i) Based on all genomes in the training set, Illumina SE75 reads of a gradient genome sequencing depth (0.05×, 0.1×, 0.2×, 0.3×, 0.4×, 0.5×, 0.6×, 0.7×, 0.8×, 0.9×, 1×, 2×, 3×, 5×, 10×, 30×) were simulated to evaluate the effect of the amount of sequencing data and to set mNGS-AST reporting rules. First, a line chart of the AUC value was drawn, and the lowest sequencing depth was determined when the AUC value tended to be stable for each model. A score value that met PPV ≥ 90% was set as the threshold for reporting “R”, while a score value that met a higher negative predictive value (NPV), such as above 90%, was set as the threshold for reporting “S” only when the amount of sequencing data was above the lowest sequencing depth (more details can be found in Section 6 of the supplemental material).

(ii) Simulation testing of coinfected bacteria was performed to assess the accuracy of the species attribution of the ARGs. Illumina SE75 reads from 30 A. baumannii strains with the OXA-23 gene (without NDM) and 30 Klebsiella pneumoniae strains with the NDM gene (without OXA-23) were simulated and gradiently mixed together to perform the read-based ARG test for assessing the accuracy of ARG species attribution (see Section 6 in the supplemental material).

### Validation and application of the mNGS-AST model using clinical specimens.

A total of 230 clinical specimens, including 122 bronchoalveolar lavage fluid (BALF), 90 sputum, 4 cerebrospinal fluid, 3 ascites, and 11 other samples with positive A. baumannii cultures from 220 patients were collected retrospectively from Guangdong Provincial People's Hospital and Tianjin Medical University General Hospital from October 2020 to September 2021. The clinical isolates were identified using a Bruker Microflex MALDI-TOF MS (Bruker Daltonics, Bremen, Germany) with scores of more than 2.0, and clinical specimens were sequenced using mNGS by Genskey Medical Technology. DNA from all samples was extracted using the TIANamp Micro DNA kit (TIANGEN) and quantified by fluorometry (Thermo Fisher Scientific). DNA libraries were generated using an NGS library construction kit (Enzymatics) with unit dual index adapters. Samples with a DNA concentration of <1 ng/μL were subjected to PCR with the following program: initiation at 95°C × 3 min; then 5 or 10 cycles of 98°C for 20 s, 60°C for 15 s, 72°C for 30 s, and a final extension of 72°C for 5 min. The libraries were pooled to be sequenced on Illumina NextSeq 550 sequencers using a 75-cycle or 50-cycle single-end sequencing strategy. Before the read-based ARG test was conducted and the antibiotic resistance and susceptibility prediction pipeline was used, the sequencing data obtained from each specimen were subjected to quality control and host nucleic acid sequence filtering. Afterward, pathogen identification via the bioinformatics pipeline was performed as previously described (31). Finally, PPV, negative predictive value (NPV), and the proportion of effective reportable samples were determined to evaluate the performance of the mNGS-AST model. PPV refers to the probability that an isolate is phenotypically resistant when genotypic resistance is predicted by mNGS. Similarly, NPV is the probability that an isolate is phenotypically susceptible when genotypic susceptibility is predicted by mNGS.

Another cohort of 50 clinical samples in which A. baumannii was detected by mNGS, as part of routine clinical testing for pathogen identification, were collected prospectively from Guangdong Provincial People's Hospital from November 2021 to September 2022 and cultured in parallel. The cultured suspected pathogenic bacterial cultures were tested for susceptibility using a Vitek 2 Compact. The established mNGS-AST model was used to forecast susceptibility findings, which were compared to the results of cultured AST to further evaluate the clinical application of the mNGS-AST prediction model.

### Data availability.

The genomes of 158 Acinetobacter baumannii isolates from local hospital have been deposited in NCBI under BioProject number PRJNA942016.

## RESULTS

### Genetic diversity and resistance distribution characteristics.

After low-quality strains were removed, a total of 1,942 A. baumannii strains with confirmed AST conclusions, such as “resistant” or “susceptible,” were eventually collected. The effective numbers of strains shown to be resistant or susceptible to IPM, CAZ, CPM, and CIP were 1,757, 1,472, 853, and 1,674, respectively. Among all 1,942 strains, 1,784 strains from public sources were collected mostly from the United States, while a small portion were collected from Pakistan, Germany, Australia, and other countries, and the antibiotic resistance phenotypes were measured by several different dilution or paper diffusion methods. For the 158 A. baumannii strains from domestic hospitals, WGS was performed to generate genome sequences via assembly, and AST results were measured by the Vitek-2 system (see Tables S1 and S2 in the supplemental material).

As shown in the maximum likelihood phylogenetic tree, these strains presented a diverse substructure within the A. baumannii population, suggesting strong representation and genetic diversity (see Fig. S3A in the supplemental material). Moreover, a single subgroup entirely comprising domestic strains was clearly formed, indicating that the species has regionally distinct distributions in China. Based on the AST phenotypes, the percentage of resistance were 51.85%, 81.05%, 77.73%, and 84.65% for IPM, CAZ, CPM, and CIP, respectively. The resistance distribution was correlated with the population structure with one exception, i.e., IPM-resistant and IPM-susceptible strains emerged alternately within a subgroup. The observed different drug susceptibilities within the subgroup of strains may be attributed to the presence of mobile genetic elements of NDM ARGs, such as plasmids, insertion sequences, and transposons.

### Selection of key resistance features using the WGS-based LASSO regression model.

According to the MLST results, 1,942 strains were randomly divided into a training set (*n* = 1,557) and a test set (*n* = 385) at a ratio of 8:2 based on stratified sampling. To select the resistance features and determine their weight coefficients, a WGS-based LASSO regression model was used on the training set to investigate the association between the genetic data and phenotypic AST results. The score index was calculated based on the screened critical resistance traits (presence or absence of ARGs or ARG mutations) with weight coefficients, and the ROC curve was drawn to evaluate the model's performance.

**FIG 2:**
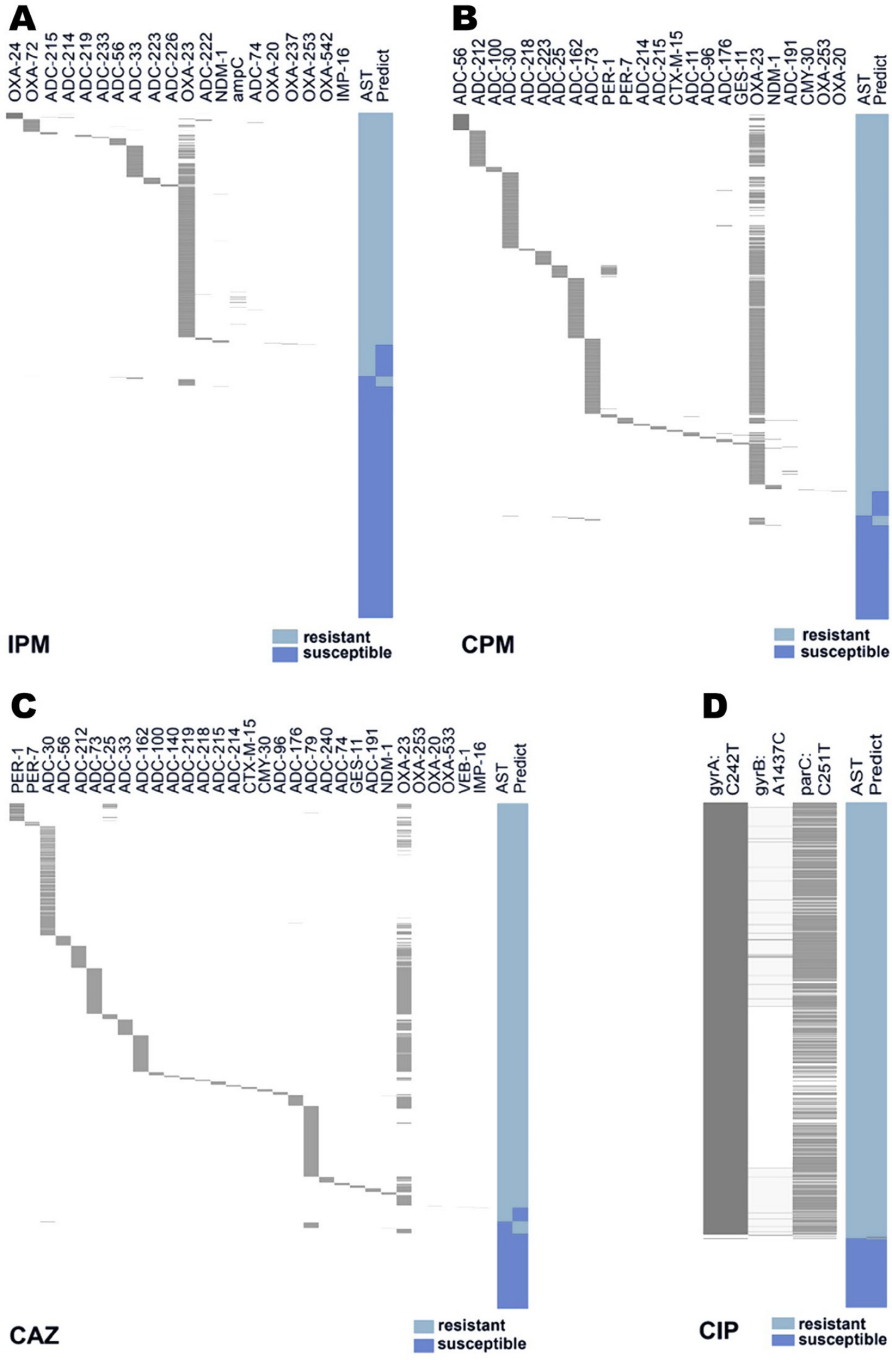
Machine learning to screen key antimicrobial resistance (AMR) features based on the WGS-AST model. (A to D) Heatmap of key AMR features detected in training samples of IPM (A), CPM (B), CAZ (C), and CIP (D). The presence of AMR features in each strain is shown in the left panel. The results of routine AST and WGS-AST are shown in the right panel. Gray, detected features; cyan, resistant; royal blue, susceptible.

The numbers of screened resistance signatures of A. baumannii for IPM, CAZ, CPM, and CIP were 20, 31, 24, and 3, respectively. For IPM, the antimicrobial resistance (AMR) signatures included *OXA-23*, *OXA-24*, *OXA-72*, *NDM-1*, *IMP-16*, some *ADC* subtypes, and some *OXA* subtypes with low frequency. The AUC values were 0.93 and 0.94 in the training and test sets, respectively (Fig. 2A; Table 1). The AUC values of the model of CPM, CAZ, and CIP were all greater than 0.95 in the training and test sets (Fig. 2B to D; Table 1). The weight coefficients of these resistance features are listed in Table S4 in the supplemental material for the four most important antibiotics.

**TABLE 1:** Performance of the LASSO regression classifier to predict A. baumannii susceptibility or resistance to four antibiotics^*a*^

Antibiotic	Cutoff	No. of true resistance	No. of false resistance	No. of true susceptibility	No. of false susceptibility	Sensitivity	Specificity	PPV (%)	NPV (%)	Accuracy (%)	AUC (Tr|Va)^*b*^	Type of markers^*c*^	No. of markers^*d*^
IPM	2.33	649	29	646	87	0.88	0.96	0.96	0.88	0.92	0.93 | 0.94	GPA	20
CAZ	2.27	947	28	177	33	0.97	0.86	0.97	0.84	0.95	0.97 | 0.96	GPA	31
CPM	1.74	500	13	124	32	0.94	0.91	0.97	0.79	0.93	0.95 | 0.96	GPA	24
CIP	2.00	1,161	2	184	4	1.00	0.99	1.00	0.98	1.00	0.99 | 0.99	SNPs	3

a True resistance, phenotypical AST is resistant and genotypic resistance is predicted. False resistance, phenotypical AST is susceptible and genotypic resistance is predicted. True susceptibility, phenotypical AST is susceptible and genotypic susceptibility is predicted. False susceptibility, phenotypical AST is resistant and genotypic susceptibility is predicted.

b Tr, Training set; Va, validation set.

c GPA, gene presence or absence; SNPs, single nucleotide polymorphisms.

d The number of features determined when AUC value is higher or cross-validation error is small.

Furthermore, subsets of different sample sizes with 10 replicates were randomly selected for LASSO regression analysis to assess the impact of sample size on the performance of the predictive model. As shown in Fig. S3B, when the sample size was more than 300 cases, the performance of each model tended to be stable.

### The mNGS-AST model established based on short-read alignment and evaluated in simulated experiments.

Based on the selected resistance features, a read-based software process was developed using mNGS data to determine the presence or absence of ARGs and their mutations to infer the species attribution of ARGs and predict the antibiotic susceptibility information. More details are shown in Fig. S2.

**FIG 3:**
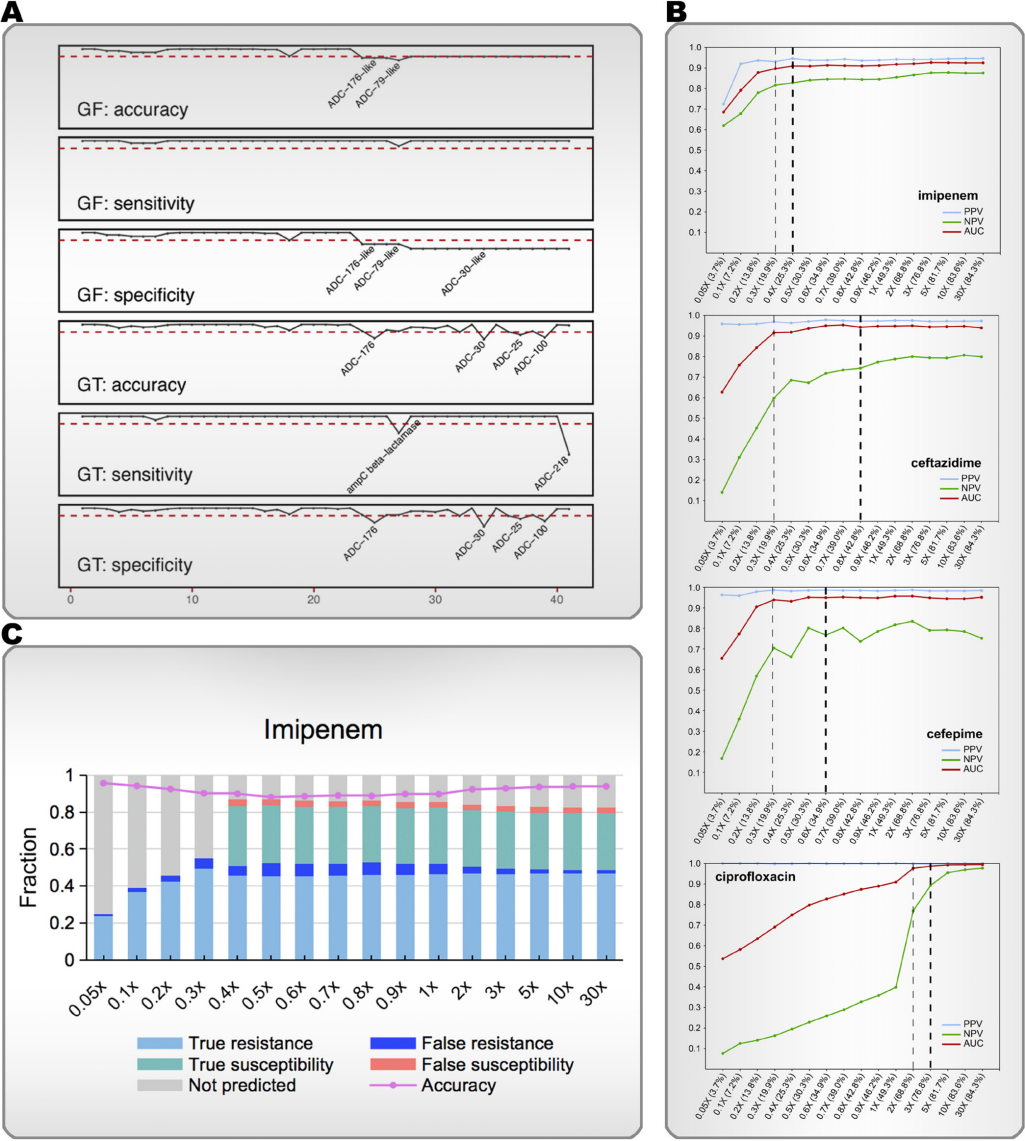
Simulation test to determine the reported cutoff value and evaluate the performance of the mNGS-AST prediction model. (A) Performance of the read-based gene test module, as measured by an assembly-based pipeline with sufficient data amount (simulated 30× sequencing depth) produced from the simulation experiment. Gene families or gene types with accuracy, sensitivity, or specificity <0.9 are shown in the graph. GF, gene family; GT, gene type. (B) Read-based classification performance at different average sequencing depths. The abscissa is the amount of data with different sequencing depths, and average genome coverage is recorded in brackets. The AUC plateaus when the sequencing depth is >0.3× in the imipenem, ceftazidime, and cefepime models and >2× in the ciprofloxacin model. The minimum sequencing depths required for reporting susceptibility were 0.4×, 0.8×, 0.6×, and 3× for the imipenem, ceftazidime, cefepime, and ciprofloxacin models, respectively. (C) Imipenem read-based model performance at different sequencing depths. The abscissa is the amount of data with different sequencing depths. The bars show the rate of true resistance (AST-R/predict-R), false resistance (AST-S/Predict-R), true susceptibility (AST-S/Predict-S), false susceptibility (AST-R/Predict-S), and unpredictable samples. The broken line depicts the predictive accuracy of predictable samples.

To evaluate the performance of the read-based ARG detection and susceptibility prediction process, short-read sequences with different amounts of sequencing data were simulated based on the strain genome using the training set. The accuracy of ARG subtyping, except for some *ADC* subtypes, was mostly above 0.9 when compared with the assembly-based test results, and the AUC value of each prediction model was above 0.9 when the amount of sequencing data was sufficient (such as 30× sequencing depth) (see Fig. 3A; see also Table S5 in the supplemental material). The curve depicting changes in the AUC value was used to determine the minimum amount of sequencing data when the model tended to be stable in the analysis of resistance/susceptibility to a specific antibiotic. As shown in Fig. 3B, when the sequencing depth was 0.3× for IPM, CAZ, or CPM and 2× for CIP, the mNGS-based resistance models tended to be stable.

Given the impact of the amount of sequencing data on the model’s performance, the optimal reporting thresholds were set for predicting “resistance” and “susceptibility” to the four antibiotics according to the simulation test results with different amounts of data (see Table S6 in the supplemental material). The prediction accuracy and proportion of valid reportable samples were calculated by the prediction model using different volumes of data. The proportion of effectively reported samples increased with increasing amounts of sequencing data in the analyses of all four antibiotics. “Susceptibility” to IPM, CAZ, CPM, and CIP could not be predicted when the sequencing depth was less than 0.4×, 0.8×, 0.6×, and 3×, respectively (Fig. 3C; see also Table S7 in the supplemental material).

In addition to determining the resistance genes and mutations based on the above read-based prediction procedure, it was important to correctly predict the species source of the detected ARGs. The simulation test showed that the attribution of ARG species may be properly predicted (accuracy, >0.95) when the genome sequencing depth of the target species containing resistance genes is more than 8 times higher than that of the other species, which was based on simulation data using the mixed A. baumannii and K. pneumoniae strains with OXA-23 or NDM genes (see Fig. S4 in the supplemental material).

### Clinical validation and application using clinical specimens.

**FIG 4:**
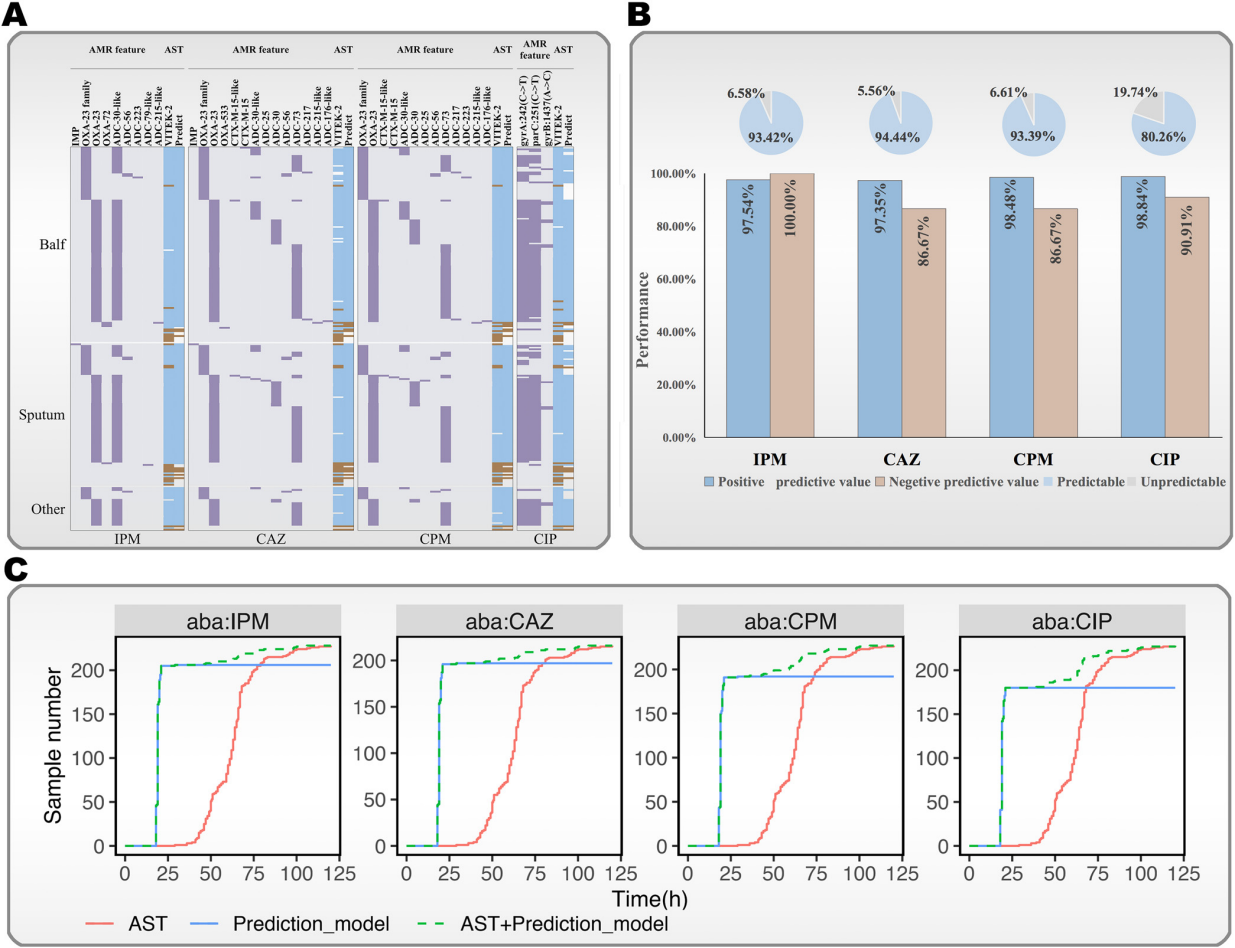
mNGS-AST model for predicting the antibiotic resistance susceptibility of A. baumannii directly from clinical samples. (A) Prediction of the resistance and susceptibility of A. baumannii to CAZ, CIP, CPM, and IPM using 230 clinical samples. The left panel indicates the presence of key AMR features, while the right panel compares the results of phenotypic AST and mNGS-AST. Purple, the presence of AMR features in each strain; cyan, resistant; brown, susceptible; ND, not detectable; NP, not predicted. (B) Results of statistical analysis of results shown in panel A. Pie chart, predictive ratio. (C) Comparison of the turnaround time between routine AST and mNGS-AST. Regarding predictable samples, mNGS-AST reports AMR results in approximately 19.1 h, saving an average of 44.3 h compared with routine culture-based AST. Aba, A. baumannii.

**(i) Validation of the mNGS-AST prediction model using retrospective clinical specimens.** A total of 230 clinical specimens with positive A. baumannii cultures were collected and sequenced by mNGS for validation purposes. The genome coverage of detected A. baumannii ranged from 0.11% to 86.33%. The proportion of effectively reported samples for each resistance model was 93.42% (IPM), 94.44% (CAZ), 93.39% (CPM), and 80.26% (CIP). Our method classified susceptible or resistant phenotypes with an accuracy of 97.65% for IPM, 96.57% for CAZ, 97.64% for CPM, and 98.36% for CIP. The PPVs were all greater than 0.97. The NPVs for predicting “susceptibility” were 100% for IPM, 86.67% for CAZ, 86.67% for CPM, and 90.91% for CIP (Fig. 4A and B and Table 2; see also Table S8 in the supplemental material).

**TABLE 2:** Performance of the mNGS-AST model to predict susceptibility and resistance to four antibiotics in 230 clinical specimens^*a*^

Antibiotic	No. of true resistance	No. of major error	No. of true susceptibility	No. of very major error	Accuracy (%)	PPV (%)	NPV (%)	Predictable ratio (%)	AST_sample_n	Predict_sample_n
IPM	198	5	10	0	97.65	97.54 (198/203)	100.00 (10/10)	93.42 (213/228)	RS:228 (R:202-S:24-I:2)	RS:213 (R:203-S:10)
CAZ	184	5	13	2	96.57	97.35 (184/189)	86.67 (13/15)	94.44 (204/216)	RS:216 (R:189-S:26-I:1)	RS:204 (R:189-S:15)
CPM	194	3	13	2	97.64	98.48 (194/197)	86.67 (13/15)	93.39 (212/227)	RS:227 (R:186-S:25-I:16)	RS:212 (R:197-S:15)
CIP	170	2	10	1	98.36	98.84 (170/172)	90.91 (10/11)	80.26 (183/228)	RS:228 (R:203-S:25-I:0)	RS:183 (R:172-S:11)

a AST_sample_n, the number of samples with AST conclusion such as resistance or susceptibility. Predict_sample_n, the number of samples with prediction results such as resistance or susceptibility. Predictable ratio, the ratio of Predict_sample_n to AST_sample_n. Major error, phenotypical AST is susceptible and genotypic resistance is predicted. Very major error, phenotypical AST is resistant and genotypic susceptibility is predicted.

We evaluated the time-effectiveness of the mNGS-AST prediction model and routine culture-based phenotypic AST using these 230 samples. The mNGS-AST model showed a significantly shorter turnaround time of approximately 19.1 h to report AMR results in contrast to the culture-based approach, which required 66.3 h (Fig. 4C; see also Table S9 in the supplemental material). Susceptibility reports will be timelier and more effective if the two methods are integrated.

**FIG 5:**
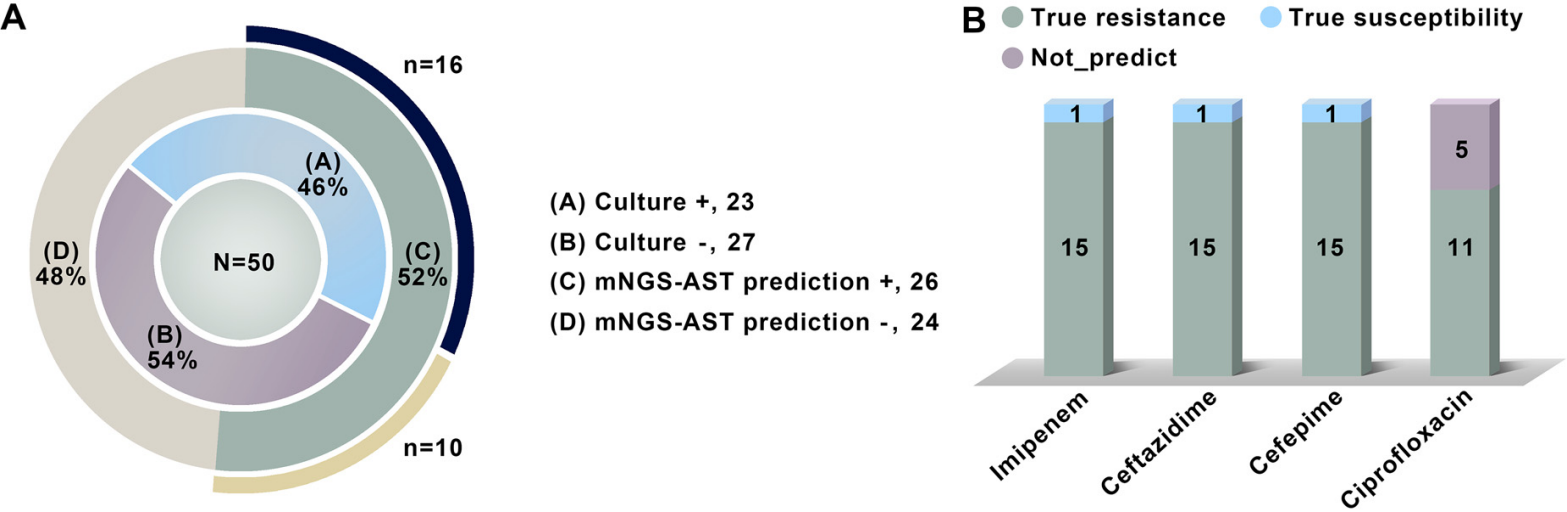
Comparison of mNGS, mNGS-AST prediction, and culture with phenotypic AST results for A. baumannii in prospective samples. The inner ring shows the distribution of culture-based AST, while the outer ring indicates mNGS-AST prediction results. More details can be found in Table S10 in the supplemental material. (A) Statistics of mNGS-AST prediction (the outer ring) versus culture-based AST results (the outer ring) in 50 samples. Culture +, culture is positive; Culture −, culture is negative; mNGS-AST prediction +, resistance or susceptibility cannot be predicted by the mNGS-AST model. The outermost black arc represents the overlap between Culture + and mNGS-AST prediction +, while the orange arc represents Culture − and mNGS-AST prediction +. (B) Consistency between mNGS-AST prediction and culture-based phenotypic AST results in 16 samples. True resistance, phenotypical AST is resistant and genotypic resistance is predicted. True susceptibility, phenotypical AST is susceptible and genotypic susceptibility is predicted.

**(ii) Application of the mNGS-AST prediction model using prospective clinical specimens.** Fifty A. baumannii-positive clinical samples detected by mNGS were compared with culture results to further verify the developed models. As shown in Table S10 in the supplemental material and Fig. 5, almost all culture results were found in the mNGS results, indicating the good diagnostic consistency and performance of mNGS. The prediction models yielded 26 samples with clear indications of drug resistance (R) (*n* = 20) or sensitivity (S) (*n* = 6), and the consistency with the available phenotypic AST results (*n* = 16) was 100% (16/16; S or R). mNGS-AST models successfully predicted the AST results for 10 culture-negative samples, thereby potentially providing additional information for clinical medication and suggesting the feasibility of the model for use in culture-negative patients. Additionally, among the 20 samples with indications of drug resistance, 15 A. baumannii samples were found to be pandrug resistant based on the phenotypic AST results (only susceptible to tigecycline and polymyxin B). Moreover, 6 samples were projected to be sensitive to IPM, CAZ, and CPM, providing evidence that these were clinically treatable A. baumannii infections. Polymyxin B, tigecycline, or other combination therapies should be considered immediately if the predicted AST results are all resistant. The fact that the mNGS model predicted susceptibility (1 day) considerably faster than traditional AST (2 to 3 days) indicates that this approach could save valuable time during clinical treatment.

## DISCUSSION

In this study, we established a read-based AST prediction approach to identify A. baumannii and directly predict its resistance and susceptibility to imipenem, ceftazidime, cefepime, and ciprofloxacin. Notably, targeted resistance features were precisely selected by using a machine learning algorithm with large-scale genomes and susceptibility results instead of directly using antibiotic-related resistance genes issued by public databases, thereby making the model resistance targets more accurate. Simulation trials and clinical samples both showed that the prediction accuracy of our model was above 95% compared with culture-based AST results. These prediction models, therefore, could facilitate the diagnosis of A. baumannii infection and may be used as a rapid AST method in clinical practice.

The accuracy of resistance signatures screened by machine learning largely depends on the representativeness and genetic diversity of the data set. In this study, the utilized strains consisted of 1,784 genomes from PATRIC and NCBI NDARO and 158 strains from domestic hospitals, maximizing the generalization capacity of the model during establishment of the classifier model. The genetic diversity and regional specificity of the strains were also revealed by phylogenetic analysis in accordance with previous studies (32). Our prediction models for imipenem, ceftazidime, cefepime, and ciprofloxacin all had an accuracy of over 0.92. Therefore, the procedures used to screen key resistance characteristics and to generate the model are feasible, establishing the groundwork for the generation of a short-read-based alignment model.

The current A. baumannii resistance prediction model predicts AMR based on WGS data since those obtained by WGS are comprehensive and accurate data (19). Despite their excellent performance, WGS-based techniques are limited by high financial costs and inaccessibility in resource-poor settings, making them difficult to implement in clinical practice. With the popularization of mNGS technology, we innovatively developed an AST prediction model based on short-read alignment and a machine learning algorithm, enabling the prediction of antibacterial phenotypes directly using clinical samples in less than 24 h, which provides a clear advantage over culture-based AST. The PPVs observed in the analysis of imipenem, ceftazidime, cefepime, and ciprofloxacin resistance/susceptibility were all over 97%, and the proportions of effectively reported samples were 93.42%, 94.44%, 93.39%, and 80.26%, respectively. Recent efforts have focused on analyzing ARGs and establishing prediction models using mNGS to directly predict antibacterial phenotypes. mNGS has been used for the diagnosis of pathogens, and the feasibility of using mNGS read-based detection for ARGs of Klebsiella aerogenes, Streptococcus, and *Actinomyces* in clinical specimens has been evaluated (33, 34). A genomic neighbor-typing method was used to directly infer the antibacterial phenotype for Streptococcus pneumoniae based on nanopore sequencing sputum, with a sensitivity and specificity of 75% and 100%, respectively (35). Corroborating recent mNGS studies, our investigation provides new insight and evidence that mNGS-AST could serve as a novel rapid AST detection method for clinical precision.

Our study had several limitations. First, in clinical specimens that are concurrently infected or colonized with other bacteria at an abundance that is comparable to that of A. baumannii, the identified ARGs may not be accurately attributed to the target species; therefore, the prediction module used to identify the species source of genes still needs further optimization. Second, due to the complexity of clinical samples and shallow depth of sequencing data obtained from pathogenic bacteria resulting from low microbial biomass and high host DNA content, the resistance or susceptibility may not be effectively determined using the model when the detected amount of sequencing data does not reach the minimum amount required for model stability (such as genome coverage of target pathogens > 30%). Particularly, for predicting sensitivity, to ensure the accuracy of the prediction results (usually >0.9) and minimize the probability of very major errors, the drug susceptibility was mostly reported in this work; therefore, genotypic susceptibility results for some clinical samples with sequencing data below the threshold amount could not be obtained (see Table S10 in the supplemental material). In addition, this study only focused on A. baumannii resistance/susceptibility to four first line antibacterials. This strategy and model could be generalized to analyze a wider range of antibacterials and clinically infectious pathogens, such as S. aureus, E. coli, K. pneumoniae, Enterococcus faecalis, Enterococcus faecium, and Pseudomonas aeruginosa.

In summary, we developed and validated an mNGS-based model that can be used to diagnose A. baumannii and predict antibacterial phenotypes in less than 24 h. This method could provide a good basis for early drug recommendations while clinicians wait for the antibacterial phenotype using the traditional culture-based AST methods and merits further investigation.
