# Supplementary material for: Novel Clinical mNGS-Based Machine Learning Model for Rapid Antimicrobial Susceptibility Testing of Acinetobacter baumannii
Source: J Clin Microbiol. 2023 Apr 6;61(5):e01805-22. doi: 10.1128/jcm.01805-22 (PMC10204632; doi:10.1128/jcm.01805-22)
Supplement: Supplemental file 1 — Supplemental material. Download jcm.01805-22-s0001.pdf, PDF file, 0.8 MB [file jcm.01805-22-s0001.pdf]

Supplementary Materials: A novel clinical mNGS-based machine learning model for rapid antimicrobial susceptibility testing of *Acinetobacter baumannii*

Xuejiao Hu[a,*], Yunhu Zhao[a,*], Peng Han[b,*], Suling Liub[a], Weijiang Liu[a], Cong Mai[c], Qianyun Deng[a], Jing Ren[d], Jiajie Luo[b], Fangyuan Chen[b], Xuefeng Jia[b], Jing Zhang[b], Guanhua Rao[b,#], Bing Gu[a,#]

[a] Department of Laboratory Medicine, Guangdong Provincial People's Hospital (Guangdong Academy of Medical Sciences), Southern Medical University, Guangzhou, China

[b] Genskey Medical Technology Co. Ltd., Beijing, China

[c] Department of Critical Care Medicine, Guangdong Provincial People's Hospital (Guangdong Academy of Medical Sciences), Southern Medical University, Guangzhou, China

[d] Tianjin Medical University General Hospital, Tianjin, China

Running Head: A novel mNGS-based model for rapid AST

# Address correspondence to Bing Gu, gubing@gdph.org.cn. Guangdong Provincial People's Hospital (Guangdong Academy of Medical Sciences), Southern Medical University, NO.106 Zhongshan Second Road, Guangzhou 510080, Guangdong, China.

# Address correspondence to Guanhua Rao, gh.rao@genskey.com. Genskey Medical Technology Co. Ltd., Building 3, Bohui Innovation, No. 9 Life Science Park Road, Zhongguancun Life Science Park, Changping District, Beijing 102206, China.

* Xuejiao Hu, Yunhu Zhao and Peng Han contributed equally to this work.

Content

Supplementary Methods..3

Section 1: The customized criteria for excluding *Acinetobacter baumannii*.....................3

genomes with low quality.. 3

Section 2: Phylogenetic analysis and multilocus sequence typing (MLST)...................... 3

Section 3: Curation of the antibiotic resistance gene (ARG) reference database.............. 4

Section 4: Assembly-based ARG test using WGS data.. 5

Section 5: ARG annotation and selection for the read-based ARG test pipeline...............6

Section 6: Simulation experiment..6

Supplementary Figures...9

Supplementary Figure 1. Cluster analysis to define *ADC* subgroups associated with resistance to CAZ, CPM, and IPM... 9

Supplementary Figure 2. Flowchart of the mNGS-based AST prediction pipeline........ 10

Supplementary Figure 3. Phylogenetic tree and classification performance of training samples using *A. baumannii* strains...11

Supplementary Figure 4. Performance of predicting the species attribution of ARGs through simulation test of the mixed *A. baumannii* and *K. pneumoniae* strains.............13

Supplementary Tables...14

Supplementary Methods

Section 1: The customized criteria for excluding Acinetobacter baumannii genomes with low quality

To ensure the high quality of the collected *Acinetobacter baumannii* (*A. baumannii*) genomes, a customized criterion formulated by reference to the NCBI genome exclusion rules was applied to discard the low-quality genomes. The customized criteria for excluding genomes with low quality were as follows: a) multiple but inconsistent AST conclusions; b) genome assembly N50 < 5000 bp or number of contigs > 2000; c) assembled genome length was 1.5 times longer or 0.5 times shorter than the average length of the target species; d) the number of predicted genes was 0.5 times lower than the average number of the target species or not satisfied (0.5 ≤ predicted number of CDS/(genome length/1000) ≤ 1.5); e) average nucleotide identity (ANI) < 0.95 or the taxonomy annotation by aligning genome contigs to the NCBI NT database was not consistent with the target species; and f) 16S rDNA was not detected.

Section 2: Phylogenetic analysis and multilocus sequence typing (MLST)

Phylogenetic trees were constructed based on the single-copy core genes of *A. baumannii* to explore genetic diversity within the population. The coding genes for each strain were first predicted using GeneMarkS software (Version 4.17) (http://topaz.gatech.edu/GeneMark) [1]. Subsequently, core and specific genes were analyzed by CD-HIT (Version 4.6.1) for rapid clustering of similar proteins, with a threshold of 50% pairwise identity and 0.7 length difference cutoff in amino acids [2]. Multiple sequence alignment of single-copy genes was performed using MUSCLE software, and phylogenetic trees inferred with the maximum likelihood model were constructed using Treebest (Version 1.9.2) based on transformed CDS [3].

Evolutionary tree plots were displayed using iTOL (https://itol.embl.de) annotated with the source and AST conclusion [4].

Assembled genome contig sequences were directly aligned to all allele genes of seven housekeeping genes (*cpn60*, *fusA*, *gltA*, *pyrG*, *recA*, *rplB*, and *rpoB*) of *A. baumannii* downloaded from the pubMLST database (https://pubmlst.org) using BLASTN (Version: ncbi-blast-2.9.0+, Parameters: -evalue 1e-5 -outfmt 6 -num_alignments 10000). The hit with an exact match for each housekeeping gene was selected, and the ST was determined by comparison with the allele profiles.

1. Besemer J, Lomsadze A, Borodovsky M. GeneMarkS: a self-training method for prediction of gene starts in microbial genomes. Implications for finding sequence motifs in regulatory regions. Nucleic Acids Res. 2001;29(12):2607-2618. doi: 10.1093/nar/29.12.2607.

2. Li W, Godzik A. Cd-hit: a fast program for clustering and comparing large sets of protein or nucleotide sequences. Bioinformatics. 2006;22(13):1658-1659. doi: 10.1093/bioinformatics/btl158.

3. Edgar RC. MUSCLE: multiple sequence alignment with high accuracy and high throughput. Nucleic Acids Res. 2004;32(5):1792-1797. doi: 10.1093/nar/gkh340.

4. Letunic I, Bork P. Interactive Tree Of Life (iTOL) v5: an online tool for phylogenetic tree display and annotation. Nucleic Acids Res. 2021;49(W1):W293-W296. doi: 10.1093/nar/gkab301.

Section 3: Curation of the antibiotic resistance gene (ARG) reference database

The antibiotic resistance gene (ARG) reference database included all nucleic acid sequences from the CARD database (v 3.1.0, http://arpcard.mcmaster.ca) and two additional fluoroquinolone resistance-related wild-type genes, *gyrB* and *parC,* of *A. baumannii* downloaded from the NCBI gene database. By referring to the MEGARes database (https://megares.meglab.org), each ARG was acyclically annotated with six hierarchical level databases and by reference to the ARG family classification information from the NCBI Health database

(https://www.ncbi.nlm.nih.gov/pathogens/isolates), which enabled us to implement the LCA annotation strategy [5].

All pairwise genes in the reference database were aligned using MUSCLE software (Version 3.8.31) to calculate the similarity: similarity = total number of correctly matched bases/total global length of alignment. The cluster analysis was conducted based on the sequence similarity between any two members within the *ADC* family, and a cluster tree was plotted with the PPV annotation of CAZ, CPM, and IPM for *A. baumannii* (**Supplementary Figure 1**). Hence, *ADC* subfamilies, such as *ADC-30-like* and *ADC-240-like*, were defined.

5. eno K, Ishii A, Ito K. ELM: enhanced lowest common ancestor based method for detecting a pathogenic virus from a large sequence dataset. BMC Bioinformatics. 2014;15:254. doi: 10.1186/1471-2105-15-254.

Section 4: Assembly-based ARG test using WGS data

Assembled genome contig sequences were aligned to the curated ARG reference database using BLASTN software (Version: ncbi-blast-2.9.0+, Parameters: -evalue 1e-5 -outfmt 0 -num_alignments 10000) to test the presence or absence and SNPs/InDels of antibiotic resistance genes by parsing the m0-format alignment result with a self-built Perl script program. Only hits with identity ≥ 90% and subject coverage ≥ 60% were retained, in which the test performance was verified with a consistency of above 0.95 by comparison to annotation records from the NCBI NDARO database. For each contig's alignment regions, the hit with the highest value (hit_score*subject_coverage) was selected as the best and was annotated. Meanwhile, the copy number of detected ARGs was counted with the following formula: ARG copy number = (coverage of target ARG* depth of cover regions)/(coverage of strain genome * depth of genomic cover regions).

Section 5: ARG annotation and selection for the read-based ARG test pipeline

The ARG annotation and subtyping pipeline was developed to correctly characterize ARG subtypes by directly aligning unassembled read sequences to the ARG reference database using BLASTN software (parameters: -evalue 1e-5 -outfmt 0 -num_alignments 10000) based on mNGS. Briefly, for each read's annotation, a greedy algorithm and specific alignment strategy were first applied to identify the true or false positive of ARG, and the false positive hits were filtered out. The remaining ARGs detected were then annotated using the LCA approach. In addition, due to the randomness of mNGS, ARGs with uneven coverage of the read sequence were also considered false positives and filtered out. For the ARG belonging to a variant model such as gyrA, the variant site of < 0.2 frequency was presumed to be a false result and filtered out (**Supplementary Figure 2**).

Section 6: Simulation experiment

Short read sequences were simulated using ART software (Version 2.5.8, parameters:-ssNS50 -l 75 -f 5 -nf 0 -rs 1) to conduct read-based ARG tests. For screening key ARG subtypes, Illumina SE75 reads of different data amounts were simulated to evaluate and summarize the normal range of uniformity coverage. The threshold of uniformity coverage was determined to filter out false positive results.

1. Simulation test for mNGS-AST reporting rules.

For all strains in the training set, Illumina SE75 reads of a gradient genome sequencing depth (0.01x, 0.02x, 0.03x, 0.05x, 0.1X, 0.2x, 0.3x, 0.4x, 0.5x, 0.6x, 0.7x, 0.8x, 0.9x, 1x, 2x, 3x, 5x, 10x, 30x) were simulated to evaluate the effect of the amount of sequencing data on the performance of the prediction model and to determine the lowest sequencing depth in each model. The test performance of read-based ARG detection was assessed under 30x sequencing depth using sensitivity, specificity, and accuracy calculated by referring to the assembly based ARG test

result. The AUC value was calculated, and a line chart was drawn to assess the performance under different sequencing data sizes for each prediction model. The lowest sequencing depth could be determined when the AUC value became stable first with increasing data size for each model.

Given the data size of sequencing and performance of the prediction model, the thresholds could be set for reporting “resistant” and “susceptible”, respectively. The threshold for reporting “resistant” was determined under sufficient sequencing data size when the Youden index was max (recorded as R_ cutoff). The threshold of reporting “susceptible” was set by two criteria. On the one hand, R_cutoff was used as the threshold of reporting “susceptible” (recorded as S_Cutoff2), and the NPV must be large (usually >= 0.9). Otherwise, “susceptible” cannot be reported. On the other hand, the maximum score value with higher NPV (usually >= 0.9) was recorded as the threshold of reporting “susceptible” (recorded as S_cutoff1) under the minimum data amount of sequencing depth (recorded as gf_msd1), which must be less than or equal to S_cutoff2. Whether S_cutoff2 was suitable for the other simulated data sizes between gf_msd1 and 30X also needs to be assessed. Another minimum data size that met the designated NPV threshold (such as 0.9) was located and recorded as gf_msd2. In summary, the reporting rules for predicting resistance and susceptibility to an antibiotic were as follows:

a) If the score value was >= R_cutoff, it was reported as “resistant”.

b) If the genome coverage of the pathogen was >= gf_msd2 and the score value was less than S_cutoff2, it was reported as “susceptible”.

c) If the genome coverage of the pathogen was >= gf_msd1 && < gf_msd2 and the score value was < S_cutoff1, it was reported as “susceptible”.

d) If the genome coverage of the pathogen was >= gf_msd1 && < gf_msd2 and the score value was >= S_cutoff1 && < S_cutoffs, it was reported as "/", namely, “not predicted”. Specifically, for a purely variation model such as CIP with a higher AUC value (usually >= 0.9) in the WGS-AST model, if all variable sites were

covered and the score value was < S_cutoff1, it was also reported as “susceptible”.

e) If the genome coverage of the pathogen was < gf_msd1 and the score was < S_cutoff1, it was reported as "/". Specifically, for a purely variation model such as CIP with a higher AUC value (usually >= 0.9) in the WGS-AST model, if all variable sites were covered and the score value was < S_cutoff1, it was also reported as “susceptible”.

2. Simulation testing of the species attribution of ARGs.

A total of *A. baumannii* strains with the OXA-23 gene (without NDM) and 30 *Klebsiella pneumoniae* strains with the NDM gene (without OXA-23) were selected from the training set and downloaded from the NCBI genome database. Illumina SE75 reads of a gradient genome sequencing depth (0.01x, 0.02x, 0.03x, 0.05x, 0.1x, 0.2x, 0.3x, 0.4x, 0.5x, 0.6x, 0.7x, 0.8x, 0.9x, 1x, 2x, 3x, 5x, 10x) were simulated based on each strain genome and were mixed together between any two *A. baumannii* and *K. pneumoniae* strains. Then, the read-based ARG test pipeline was conducted to evaluate the accuracy of ARG species attribution.

Supplementary Figures

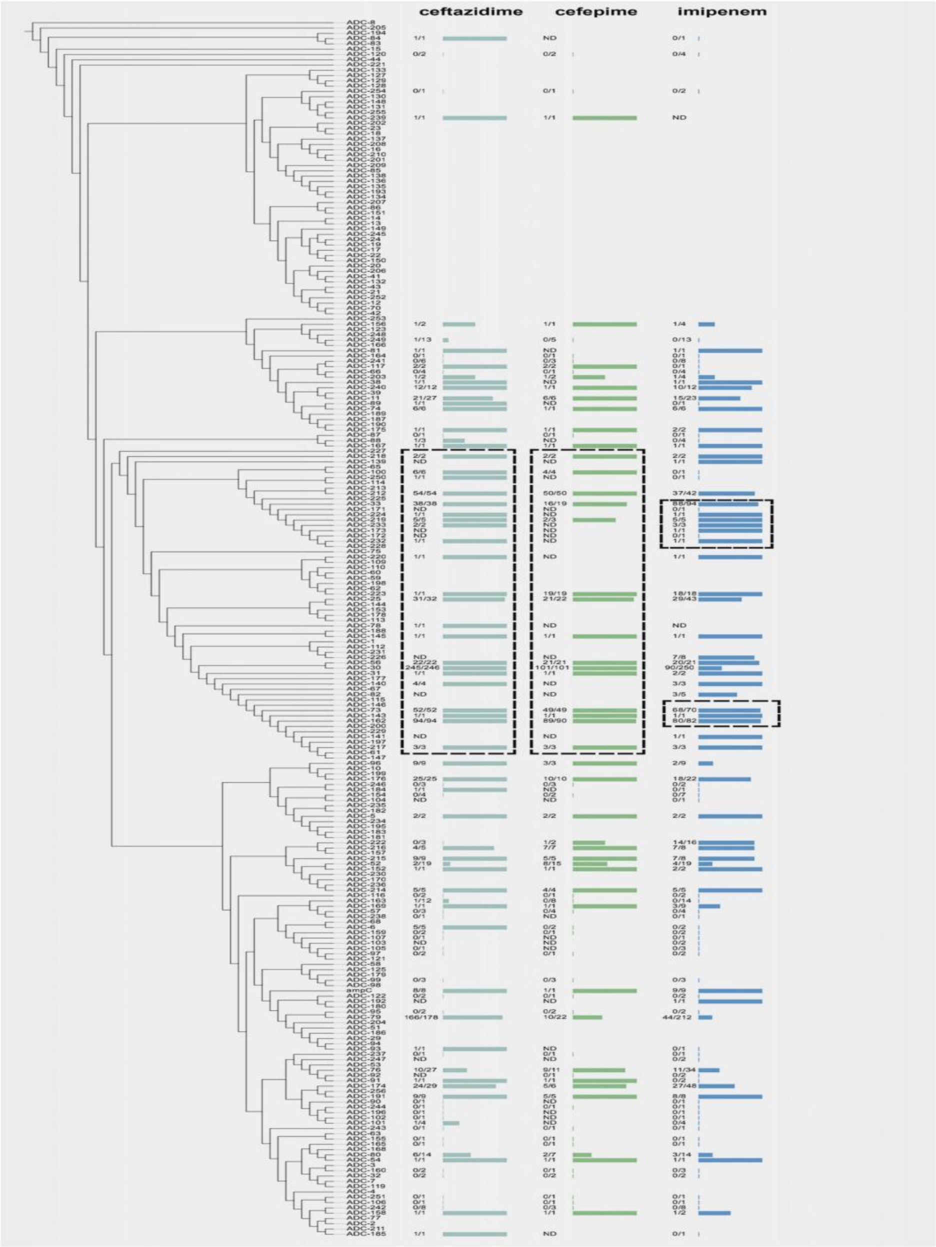

Supplementary Figure 1. Cluster analysis to define ADC subgroups associated with resistance to CAZ, CPM, and IPM. On the left, the cluster tree was plotted based on sequence identity between any two members within the *ADC* family. The PPV of each *ADC* subtype to CAZ, CPM, or IPM is displayed on the right panel, with the major *ADC* subgroups outlined with black dashed lines, such as the *ADC-30-like* family.

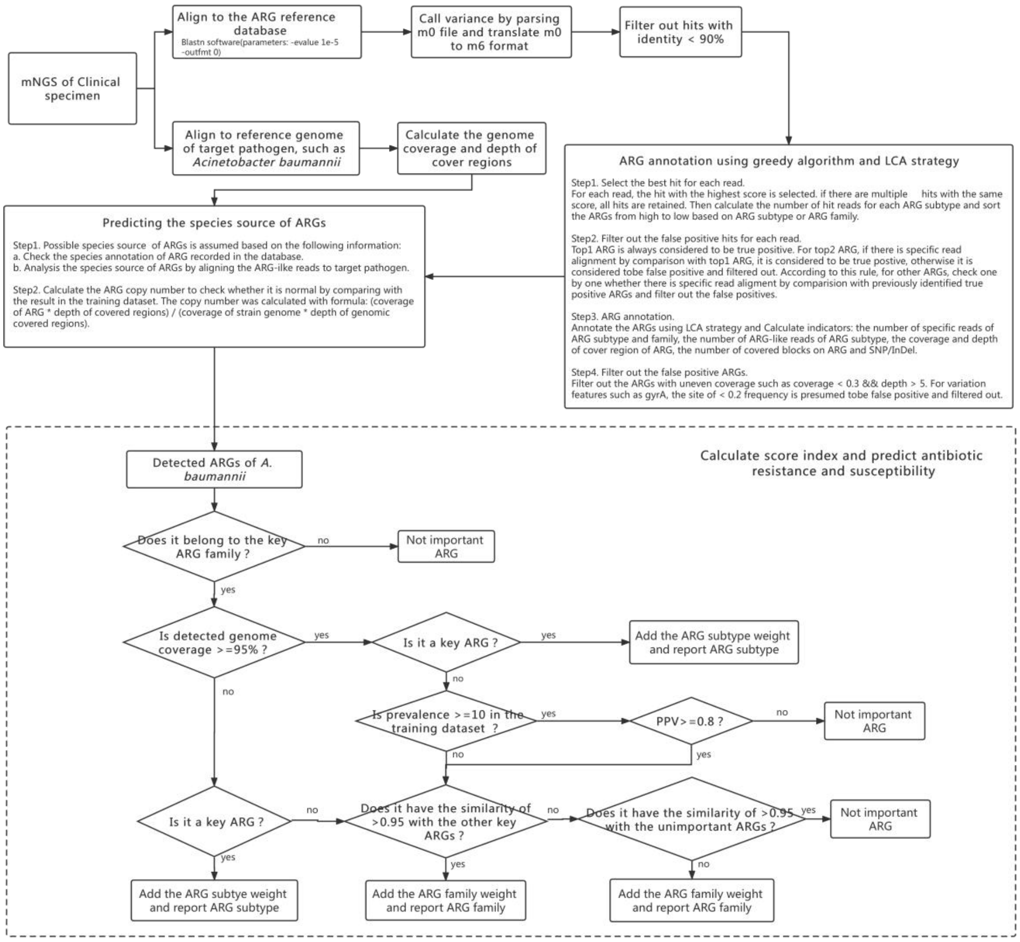

Supplementary Figure 2. Flowchart of the mNGS-based AST prediction pipeline.

The pipeline adopted a two-step strategy to accurately predict resistance and susceptibility for a pathogen-antibiotic pair. First, short reads were aligned to the curated AMR database and the reference genomes of targeted pathogens to carry out ARG annotation and species annotation and to infer possible ARG source species. Next, we predicted the probable phenotype of the sample based on the comparison of the sum score of detected ARG features with the specified cutoff value. When calculating the score index and predicting resistance and susceptibility, the weight of detected ARGs or the ARG family was added based on the result of subtyping. The prediction AST result was given by comparing the cumulative score with the threshold value.

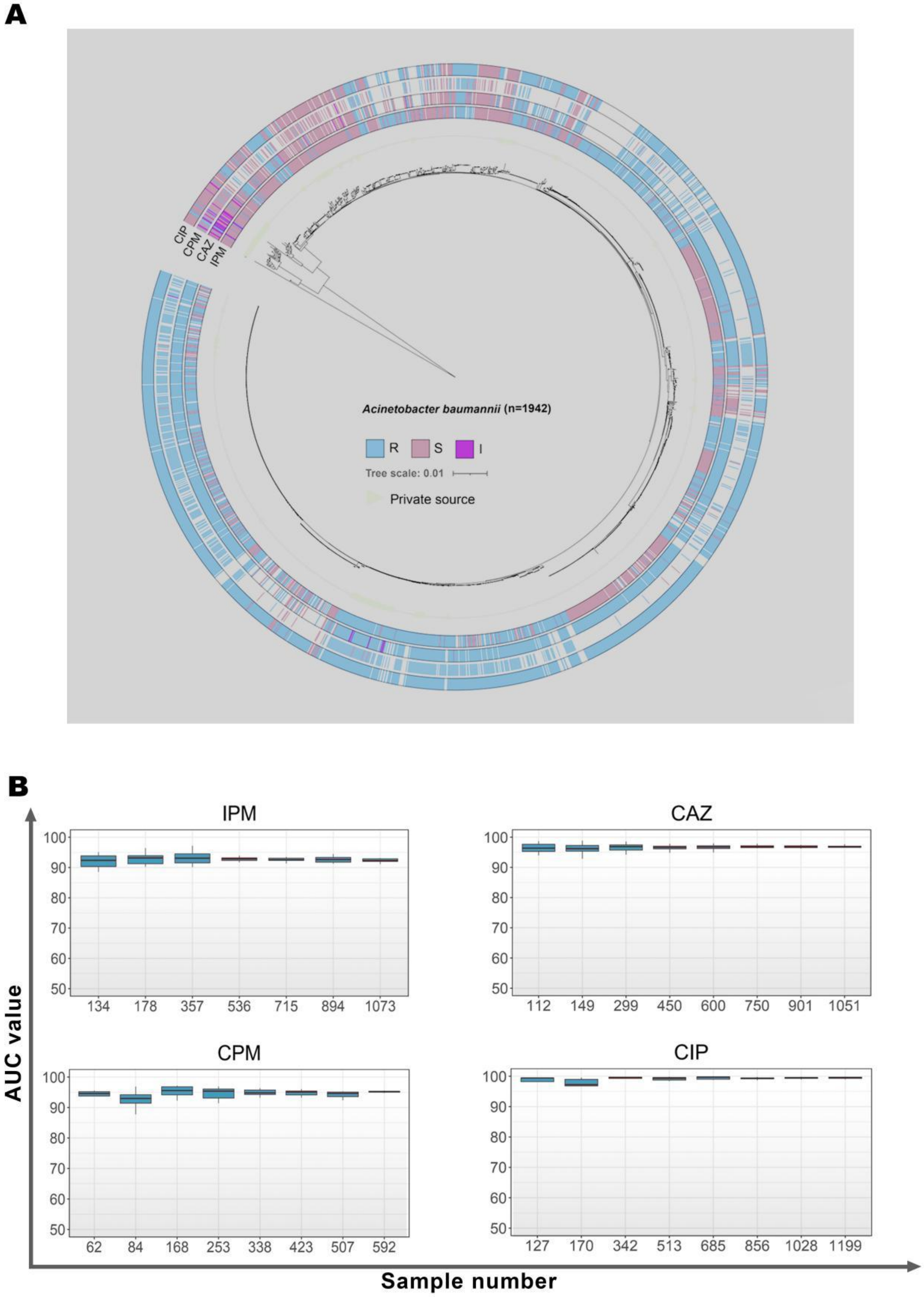

Supplementary Figure 3. Phylogenetic tree and classification performance of training samples using *A. baumannii* strains. A) maximum likelihood tree based on single-copy core genes of 1,942 *A. baumannii* strains. The blue triangle in the inner circle

represents the strains collected from domestic hospitals. The four outer circles indicate the AST conclusions of IPM, CAZ, CPM, and CIP. The phylogenetic tree revealed a high genetic diversity of the *A. baumannii* population, which makes subsequent predictive models more likely to be widely applicable. B) The classification performance improves and plateaus with increasing numbers of training samples. A lasso regression model-based resistance/susceptibility classifier was trained on various sized and randomly drawn subsamples from the training dataset and evaluated in ten repeats of a 10-fold nested cross-validation. Each panel depicts the results of the indicated antibiotics based on the presence/absence and mutants of ARG features.

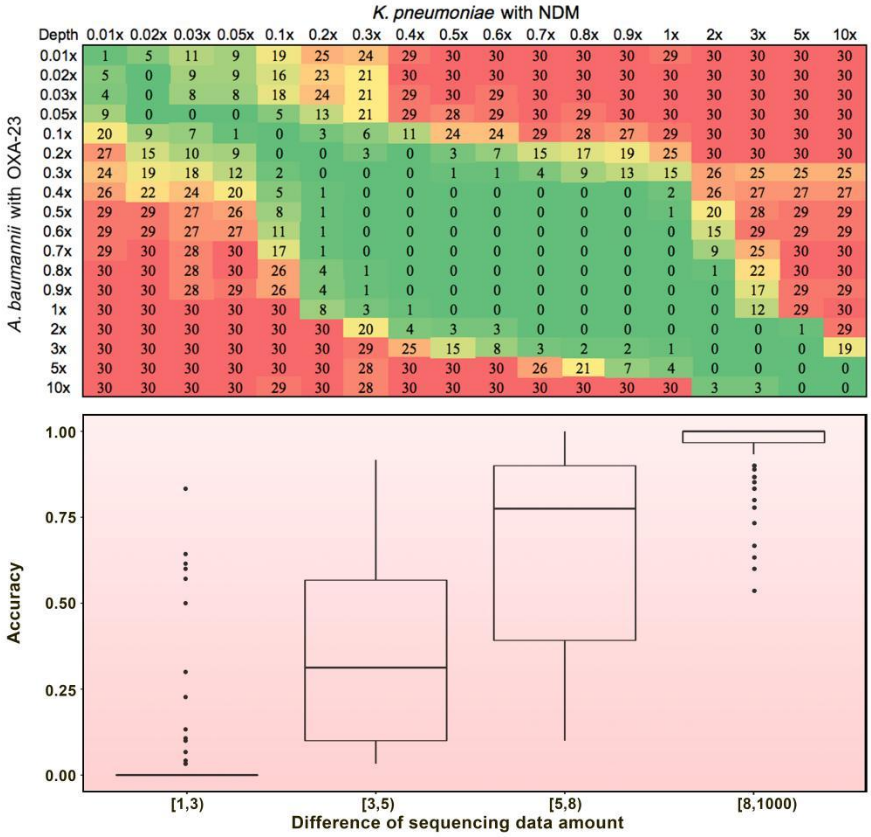

Supplementary Figure 4. Performance of predicting the species attribution of ARGs through simulation test of the mixed *A. baumannii* and *K. pneumoniae* strains. A) Heatmap with accurately assigned frequency distribution. Red denotes an accuracy of 100%, while blue indicates an accuracy of 0%. B) Boxplot shows how the performance of ARG species origins is affected by species abundance. The difference in data volume between the two species is shown on the X-axis. The Y-axis indicates the accuracy of the correct gene's species attribution. A simulation test of the mixed *A. baumannii* and *K. pneumoniae* strains showed that the abundance of *A. baumannii* carrying ARGs was at least 8 times higher than that of *K. pneumoniae*, and the attribution of OXA-23 or NDM to the species origin could be accurately predicted with an average accuracy above 0.95.

Supplementary Tables

Supplementary Table 1. Information of *A. baumannii* strain genomes downloaded from public database and collected from the Guangdong Provincial People's Hospital. Note: R, resistant; I, intermediate; S, susceptible. -, unknown. NA, not available.

Supplementary Table 2. The number of susceptible and resistant strains of *A. baumannii* used in this study. ‡Data are displayed as (Resistant|Susceptible).

Supplementary Table 3. The performance of three classifiers on different pathogen-antibiotic pairs. Note: *SD represents the variablity (standard deviation) in 10 test folds. AUC, areas under the curve; IPM, imipenem; CPM, cefepime; CAZ, ceftazidime; CIP, ciprofloxacin; LR, LASSO regression; GLM, generalized linear model; RF, random forest.

Supplementary Table 4. The contribution of selected antimicrobial resistance signatures to the LASSO regression classifier for four antibiotics in *A. baumannii*. Note: Feature PPV, positive predictive value calculated for AMR features; Gene family PPV, positive predictive value calculated for gene family which the AMR feature belongs to. *, AMR feature with PPV < 0.8 is not listed.

Supplementary Table 5. Performance of short-read (75bp)-based models to predict susceptibility or resistance to four antibiotics in simulated 30X data size. * Threshold value indicates the cut-off value that resulted in the maximal Youden index (J=sensitivity+specificity−1) for each drug.

Supplementary Table 6. Performance of short-read (75bp) based models to predict susceptibility or resistance to four antibiotics in simulated diffrerent data size. Note: Column Status, RS means resistance or susceptibility is reportable while R means only resistance is reportable. Column Cutoff (R_cutoff:S_cutoff) means the threshold to predict resistance and susceptibility separately.

Supplementary Table 7. Cut-off values for the mNGS-AST models in clinical

samples. Note: Minimum genome coverage required for susceptibility, the maximum value of truly detected genome coverage is in brackets.

Supplementary Table 8. Detailed information on read-based ARG tests to predict susceptibility and resistance to four antibiotics in 230 retrospective clinical specimens. Note: R, resistant; I, intermediate; S, susceptible. "/", not predicted or detected.

Supplementary Table 9. Turn-around time of phenotypic AST and mNGS-AST.

Supplementary Table 10. mNGS, mNGS-AST prediction and cultured *A. baumannii* information in prospective samples. Note: R, resistant; I, intermediate; S, susceptible. "/", not predicted or detected.
